# Incorporating β-Cyclodextrin with ZnO Nanorods: A Potentiometric Strategy for Selectivity and Detection of Dopamine

**DOI:** 10.3390/s140101654

**Published:** 2014-01-17

**Authors:** Sami Elhag, Zafar Hussain Ibupoto, Omer Nur, Magnus Willander

**Affiliations:** Department of Science and Technology, Campus Norrköping, Linköping University, Norrköping SE-60174, Sweden; E-Mails: zafar.hussain.ibupoto@liu.se (Z.H.I.); omer.nour@liu.se (O.N.); magnus.willander@liu.se (M.W.)

**Keywords:** ZnO nanorods, dopamine, potentiometric response, selectivity, stability, repeatability

## Abstract

We describe a chemical sensor based on a simple synthesis of zinc oxide nanorods (ZNRs) for the detection of dopamine molecules by a potentiometric approach. The polar nature of dopamine leads to a change of surface charges on the ZNR surface via metal ligand bond formation which results in a measurable electrical signal. ZNRs were grown on a gold-coated glass substrate by a low temperature aqueous chemical growth (ACG) method. Polymeric membranes incorporating β-cyclodextrin (β-CD) and potassium tetrakis (4-chlorophenyl) borate was immobilized on the ZNR surface. The fabricated electrodes were characterized by X-ray diffraction (XRD) and scanning electron microscopy (SEM) techniques. The grown ZNRs were well aligned and exhibited good crystal quality. The present sensor system displays a stable potential response for the detection of dopamine in 10^−2^ mol·L^−1^ acetic acid/sodium acetate buffer solution at pH 5.45 within a wide concentration range of 1 × 10^−6^ M^−1^ × 10^−1^ M, with sensitivity of 49 mV/decade. The electrode shows a good response time (less than 10 s) and excellent repeatability. This finding can contribute to routine analysis in laboratories studying the neuropharmacology of catecholamines. Moreover, the metal-ligand bonds can be further exploited to detect DA receptors, and for bio-imaging applications.

## 1. Introduction

Spatial and temporal analysis requirements make electrochemical sensors promising tools to investigate the role of neurotransmitters that have an electroactive nature [1–4], where the electrodes could be chemically modified for selectivity [5]. In a certain sense, miniaturized chemical sensors provide more applicable and favorable analytical methods, which have a significant attraction in the biological and chemical fields [6–8]. Since potentiometric sensors do not require external power sources and no current passes through them during detection, they are very attractive for developing sensors for biological systems. This is in addition to other advantageous features such as simplicity, cost effectiveness, and fast analysis, along with high sensitivity and selectivity [7,9].

Dopamine (DA, C_6_H_3_(OH)_2_-CH_2_-CH_2_-NH_2_) is a small and relatively simple molecule that performs diverse functions, and was identified as a potential neurotransmitter in the brain in the late 1950s by Carlsson [10]. It was found that DA receptors are implicated in many neurological processes, including motivation, pleasure, cognition, memory, learning, and fine motor control, as well as a modulation of neuroendocrine signaling. Abnormal dopamine receptor signaling and dopaminergic nerve function is implicated in several neurological disorders [11–14]. Moreover, neuroscientists were able to show that DA is found in high amounts (50 nmol/g) in a region of the brain known as the caudate nucleus. In the 1960s it was found that patients with Parkinson's disease show an almost complete depletion of DA in this region [10,15,16]. On the other hand, high levels are known to be cardiotoxic, leading to heart electrophysiology dysfunction [17]. The fact that the DA is easily an oxidizable compound makes its detection possible by electrochemical methods, i.e., using anodic oxidation. However, the majority of the previously reported electrochemical methods to determine dopamine were based on voltammetry methods and used graphene as a working electrode [1,2,18–20]. A major problem of these analyses tools is the coexistence in the biological matrix of ascorbic acid (AA) in relatively high concentrations. Usually, the concentration of DA is in the range of 10^−8^ to 10^−6^ mol·L^−1^, while that of AA in biological systems is as high as 10^−4^ mol·L^−1^ [2]. There have been few reports on the detection of DA using the potentiometric approach [21–25].

ZnO has a relatively open structure, with a hexagonal close packed lattice where Zn atoms occupy half of the tetrahedral sites. All the octahedral sites are empty, hence, there are plenty of sites for ZnO to accommodate intrinsic (Zn interstitial) defects and extrinsic dopants [26]. ZnO has been extensively used for chemical and biological sensing due to its thermal stability under usual operating conditions, as well as excellent biomimetic properties combined with high electron communication features which makes it an attractive actuator for the so-called third generation biosensors [27–33]. On the other hand, ZNRs have unique advantages in immobilizing enzymes that retain their bioactivity due to the desirable microenvironment and the direct electron transfer between the enzyme's active sites and the electrode [34–37], in addition to the relatively larger surface-to-volume ratios compared to their thin film and bulk material counterparts. It is known that the sensing mechanism of ZnO is of the surface controlled type, in which the grain sizes, surface states, and oxygen adsorption quantities all play important roles in its sensitivity [6,31,38–42].

## 2. Experimental

### 2.1. The Fabrication of ZnO NRs on the Gold Coated Glass Substrate

We have fabricated the sensor electrodes utilizing glass substrates coated with gold then followed by the growth of ZNRs. All processing steps for the preparation of the present sensor electrodes are as follows: the first step was cleaning of the substrate, where the glass substrates were sonicated in an ultrasonic bath for about 10 min in isopropanol and acetone, respectively. Then these substrates were cleaned with deionized water and lastly they were dried with an air gun. Then these glass substrates were affixed into the vacuum chamber of an evaporator instrument (Satis CR 725, Zurich, Switzerland). After this an adhesive layer of 20 nm of titanium was evaporated on the substrates and then a 100 nm thickness layer of gold thin film was evaporated.

Well-aligned ZNRs have been grown by the low temperature ACG method. This could be described as a two-step process: spin-coating a ZnO seed layer on the substrate followed by the growth of the nanorods. In the first step, the ZnO seed precursor was prepared from zinc acetate dihydrate in methanol under basic conditions as described in [43]. The solution was then spin-coated on the substrate in the first step at 1,500 rpm for 10 s and the second step at 3,000 rpm for 20 s. The second step, growth of the ZNRs, involved a hydrothermal process [44] where the substrates coated with ZnO seeds were introduced horizontally and upside-down into a 0.025 M equimolar solution of hexamethylenetetramine and zinc nitrate hexahydrate and then kept in a preheated electric oven at 90 °C for 4–6 h. Before the substrates were placed into the solution, a small part of the gold coated glass was covered in order to be used as a contact pad for the electrochemical measurements. Finally, the samples were rinsed several times in deionized water to remove any residual salt on the surface of nanostructures and then they were dried with an air gun.

The morphology and structural properties of the ZNRs was studied using a LEO 1550 Gemini field emission scanning electron microscope (Leo, Zeiss, Germany) running at 15 kV. The crystal quality of the ZnO nanorods was studied by X-ray powder diffraction (XRD) using a Phillips PW 1729 powder diffractometer (Philips, PANalytical, The Netherland) equipped with CuKα radiation (λ = 1.5418 Å) using a generator voltage of 40 kV and a current of 40 mA.

### 2.2. Membrane Preparation

The membrane is designed [21] using powdered PVC (0.18 g) which was dissolved in tetrahydrofuran (6 mL), and mixed with β-CD as ionophore (0.04 g), potassium tetrakis (4-chlorophenyl) borate as ionic additive (0.01 g), and 2-fluoro-2′-nitrodiphenyl ether (0.4 g). A stock solution containing dopamine hydrochloride (1.89 g) in deionized water (100 mL) was prepared and later diluted with a 100 mM sodium acetate-acetic acid buffer solution (pH 5.46). All reagents were analytical grade and used without any additional purification. All the chemicals were purchased from Sigma-Aldrich (Stockholm, Sweden).

**Figure 1.**
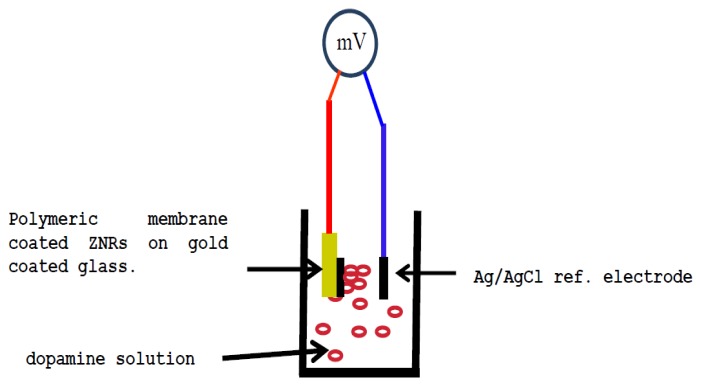
Depict potentiometric measurement for dopamine biosensor.

The as-grown ZNRs were dipped three times in the membrane solution. After that all the electrodes were left to dry in a fume hood at room temperature for one night. All the functionalized sensor electrodes were kept in a free water vapor moisture atmosphere and room temperature when not in use. The electrochemical experiments were carried using a functionalized dopamine selective sensor electrode in combination with a silver/silver chloride commercial reference electrode. The electrochemical potential was measured by using a Metrohm pH meter model 744 (Metrohm AG, Herisau, Switzerland). Figure 1 shows a diagram of the potentiometric measurement setup.

## 3. Results and Discussion

**Figure 2.**
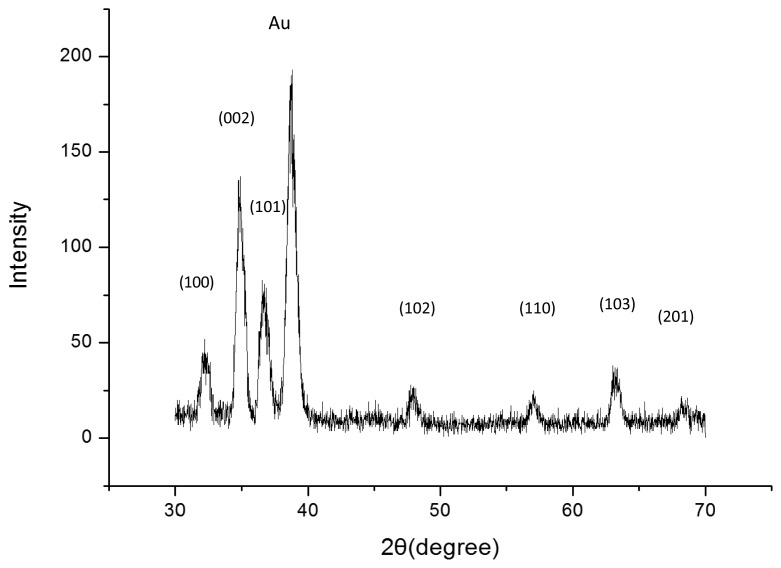
XRD spectrum of ZnO nanorods grown on a gold-coated glass substrate.

**Figure 3.**
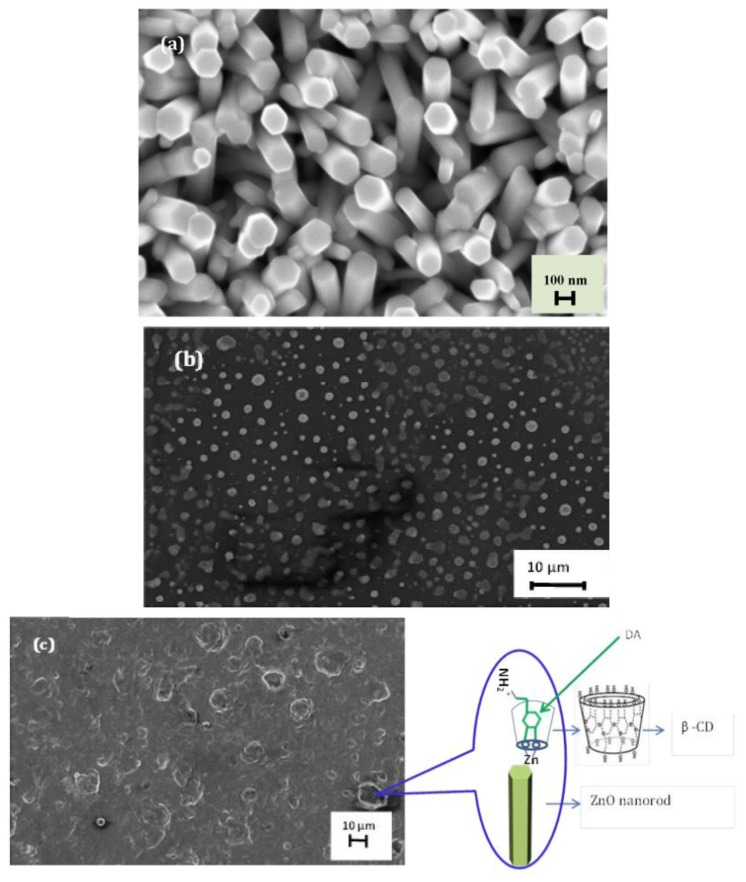
SEM images of ZNRAs grown on Au-coated glass using a low-temperature growth ACG method: (**a**) ZNRAs as grown before membrane immobilization with large aspect ratios; (**b**) and (**c**) before and after measurements respectively, zoom in, proposed mechanism for dopamine intercalation β-Cd to form metal-ligand bonds [45].

Figure 2 shows a typical XRD pattern of our sample. All the diffraction peaks of the XRD pattern can be indexed to ZnO with hexagonal wurtzite structure grown on a Au-coated glass substrate. Figure 2a shows that highly dense and well aligned grown ZNRs are grown vertically perpendicular to the surface and their shape looks like a rod with a hexagonal cross section. It can be clearly seen from SEM images that the ZNRs have diameters between 100 and 200 nm with around 1.5 μ lengths. The SEM images of the polymeric membrane film on the ZNRs before and after measurements are shown in Figure 3b,c, respectively.

**Figure 4.**
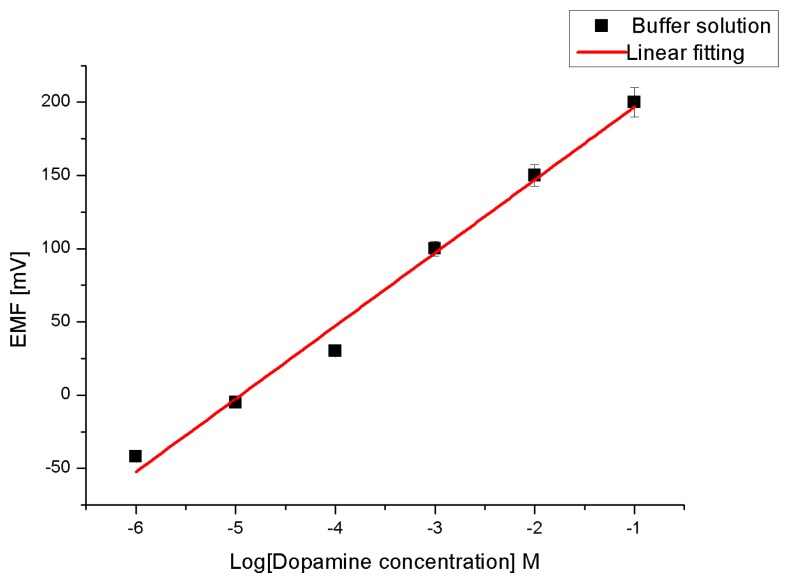
Calibration curve for the presented dopamine chemical sensor and the linear calibration equation is: y = 49.857*x* + 246.6.

Due to the high affinity of ZnO towards the dopamine molecule [46,47] to form a very strong electronic coupling (metal-ligand bond) between the ZnO and the enediol, which is a ligand hybrid system that comes from the interaction of hydroxyl groups with Zn^++^ ion, we have, therefore, prepared perm-selective membrane coated on ZNRs grown on gold-coated glass as working electrodes to endow the electrode with the property of selective transport [5,48] of dopamine molecules. To test the sensitivity of our constructed chemical sensor we performed measurements while changing the dopamine molecule concentration from 1 μM to 100 mM in the buffer solution (see Figure 4). The electromotive force is changed when the composition of the test electrolyte is modified. These changes can be related to the concentration of dopamine in the test electrolyte via a calibration procedure. However, the metal-ligand bond can be formed through a β-CD ionophore and hence the charge transfers into the buffer solution. The actual cell diagram that creates the electrochemical potential is:

[Au|ZnO buffer‖Cl−AgCl|Ag]

**Figure 5.**
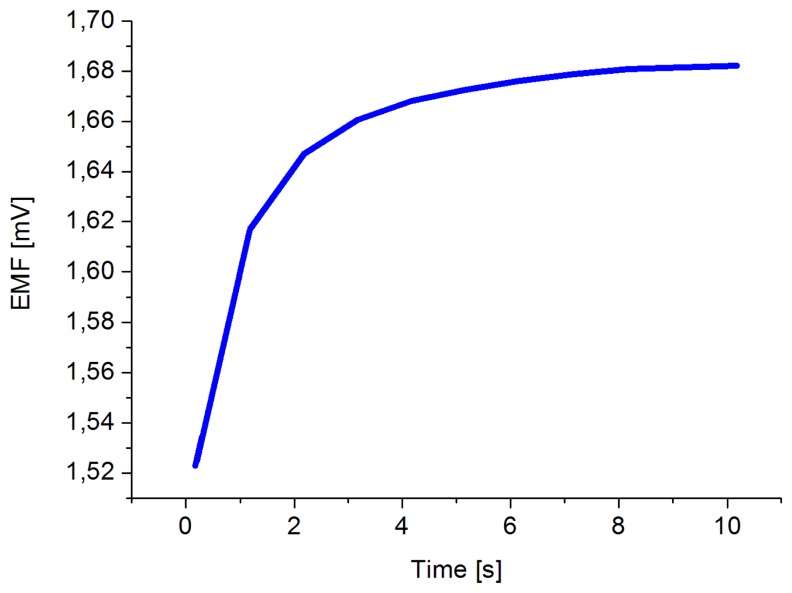
Response time measured in 0.1 mM concentration of dopamine.

The obtained results revealed that the proposed sensor possesses good linearity and sensitivity of 49 mV/decade in concentration range of 1 × 10^−6^ and up to 1 × 10^−1^ M at room temperature. Also a response time of less than 10 s was observed, as shown in Figure 5.

**Figure 6.**
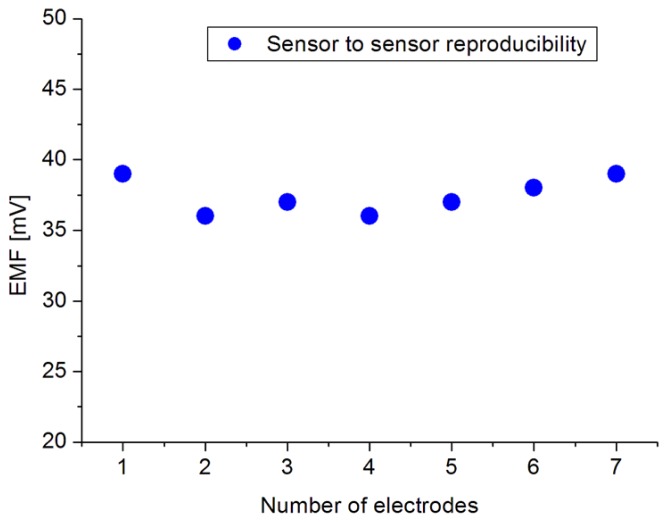
Reproducibility measured in 1 mM concentration of dopamine.

The high sensitivity and fast response time could be attributed to the high surface to volume ratio of the ZNRs which are firmly coupled with the dopamine molecules. The electrochemical sensor performance was evaluated by different important parameters [49] like the dynamic range, selectivity, detection limit, reproducibility, response time, etc. The reproducibility is a crucial property of a chemical sensor for knowing its robustness and repeatability. In order to study the reproducibility and lifetime stability of the proposed sensor, we independently synthesized seven sensor electrodes using the same set of conditions and functionalized membrane. Later, we checked the responses of all seven prepared sensors in 1 mM dopamine solution and a standard deviation of 1.35 was observed, as shown in Figure 6. The repeatability (see Table 1) has been confirmed by one sensor for three different measurements. During these three independent experiments, it was observed that the proposed sensor exhibited a maximum stable signal i.e., high sensitivity at first run of measurement after 72 h from fabrication day by maintaining the sensitivity and response time. All the measurements have been performed at a pH of around 5.5. On the other hand, the stability of the electrode i.e., lifetime, has been found to be more than 8 weeks when kept at room temperature. It can be used for this period of time without any discrepancy in the detection or sensitivity. Selectivity is the basic parameter for the description of a selective response for dopamine molecule in the presence of common interferents such as ascorbic acid, uric acid, urea and glucose. It was found that for the normal concentrations of these interferents in human serum, the presented dopamine chemical sensor showed negligible interference responses; however at a 1 × 10^−2^ M concentration of interferents a slight interference was observed. Therefore, from this we can summarize that the proposed sensor works well under the normal conditions of blood serum. Table 2 shows a comparison of the some results of the proposed DA sensor based on ZNRs with previous published sensors.

## 4. Conclusions

We have shown that ZNRs functionalized with PVC in combination with β-CD as ionophore can be used for the detection of DA by a direct physical adsorption method. It has been successfully demonstrated that the proposed sensor has a wide linear dynamic detection concentration range from 1 × 10^−6^ to 1 × 10^−1^ M for the detection of DA and a high selectivity, with no significant interference in the presence of common interfering molecules such as glucose, ascorbic acid, urea and uric acid. We believe this is due to the very strong electronic coupling between Zn^++^ ion and hydroxyl group and the pore diameter of β-CD which is ∼0.7 nm and DA is around 0.5 nm i.e., benzene ring size. The proposed sensor showed a good sensitivity of 49 mV/decade and a response time of less than 10 s. In addition, the developed sensor system functions best 72 h after from its fabrication. This time is needed when the sensor system is left to dry in air at room temperature. This finding can contribute to routine analysis in the laboratories studying the neuropharmacology of catecholamines since the principle of the developed sensor system can also extended to develop sensor systems for measuring DA receptors and bio-imaging could be investigated as well.

## Figures and Tables

**Table 1.** The repeatability response for a single electrode in three different independent experiments.

**Experiment No.**	**Linear Range (M)**	**Sensitivity (mV/decade)**	**Response Time (s)**
1	1 × 10^−6^–1 × 10^−1^	49.85	6
2	1 × 10^−6^–1 × 10^−1^	49.15	6
3	1 × 10^−6^–1 × 10^−1^	49.05	6

**Table 2.** Comparison of the result of the proposed DA sensor based on ZNRs with previous published work.

**No**	**Detection Method**	**Slope (mV/decade)**	**Time Respond (s)**	**Detection Limit (M)**	**Linear Range (M)**	**Reference**
1	Voltammetric	-	-	5.0 × 10^−8^	5 × 10^−7^–8 × 10^−4^	[18]
2	Voltammetric	-	-	-	-	[19]
3	Potentiomtric	53.3–56.2	10	6 × 10^−4^	1 × 10^−5^–1 × 10^−1^	[22]
4	Potentiomtric	59	-	-	1 × 10^−4^–1 × 10^−2^	[23]
5	Potentiomtric	59	15	8 × 10^−6^	5 × 10^−5^–1 × 10^−1^	[24]
6	Potentiomtric	30	-	5 × 10^−6^	2 × 10^−5^–1 × 10^−2^	[25]
7	Potentiomtric	49	6	1 × 10^−6^	1 × 10^−6^–1 × 10^−1^	this work
